# Hierarchical Order of Influence of Mix Variables Affecting Compressive Strength of Sustainable Concrete Containing Fly Ash, Copper Slag, Silica Fume, and Fibres

**DOI:** 10.1155/2014/646840

**Published:** 2014-02-20

**Authors:** Sakthieswaran Natarajan, Ganesan Karuppiah

**Affiliations:** ^1^Department of Civil Engineering, Regional Centre of Anna University, Tirunelveli, Tamil Nadu 627007, India; ^2^Department of Civil Engineering, Sudharsan Engineering College, Pudukkottai, Tamil Nadu 622501, India

## Abstract

Experiments have been conducted to study the effect of addition of fly ash, copper slag, and steel and polypropylene fibres on compressive strength of concrete and to determine the hierarchical order of influence of the mix variables in affecting the strength using cluster analysis experimentally. While fly ash and copper slag are used for partial replacement of cement and fine aggregate, respectively, defined quantities of steel and polypropylene fibres were added to the mixes. It is found from the experimental study that, in general, irrespective of the presence or absence of fibres, (i) for a given copper slag-fine aggregate ratio, increase in fly ash-cement ratio the concrete strength decreases and with the increase in copper slag-sand ratio also the rate of strength decrease and (ii) for a given fly ash-cement ratio, increase in copper slag-fine aggregate ratio increases the strength of the concrete. From the cluster analysis, it is found that the quantities of coarse and fine aggregate present have high influence in affecting the strength. It is also observed that the quantities of fly ash and copper slag used as substitutes have equal “influence” in affecting the strength. Marginal effect of addition of fibres in the compression strength of concrete is also revealed by the cluster analysis.

## 1. Introduction

It has been reported that manufacture of Portland cement accounts for 6-7% of total carbon dioxide emission produced by humans (http://www.builditgreen.org/). To curb further declination of environment by carbon dioxide emission, it would be better to look for materials to replace cement with suitable substitute(s) which will help in reducing the cement being used in the construction industry and thereby would reduce cement production and consumption. Production of any material mostly accompanies with wastes. While air pollution is the major concern with respect to cement production, solid waste, such as, fly ash, copper slag, and silica fume, forms the major concern in thermal power plants, copper, and silicon industries, respectively. To utilize these wastes in concrete making as replacement for cement and aggregates to certain extent would thus provide an efficient way of disposing them which would also reduce the quantity of cement being consumed in construction. It is a known fact that concrete has very poor tensile strength. To improve the tensile strength of concrete to control cracking, fibres are used.

Studies on the properties of fresh and hardened concrete utilizing fly ash, copper slag, silica fume, and fibres in concrete making have been reported widely in the literature listed in the reference. Some of the pioneering studies are briefed here. Poon et al. [1] have studied the effect of addition of large volume of low calcium fly ash in concrete as substitute for cement. They have observed that 28-day compressive strength of 80 MPa could be obtained with water cement of 0.24 and fly ash content of 45%. Also, they observed that the fly ash concrete had lesser heat of hydration and chloride diffusivity than plain cement concrete. Siddique [2] has studied the effect of replacement of cement to certain extent by fly ash in affecting the compressive strength, splitting tensile strength, flexural strength, modulus of elasticity, and abrasion resistance of fly ash concrete. It was observed that addition of fly ash decreased the performance of the concrete with respect to the parameters that have been explored. He also concludes that fly ash can be used to replace only up to 50% of cement content. Shehata and Thomas [3] have observed that increase in the content of fly ash reduced the expansion of concrete. Also, they noted that with the decrease in the silica content or with the increased content of calcium or alumina in the fly ash, the expansion increased. Malhotra [4] found that fly ash concrete had good durability with respect to frost action and low permeability of chloride ions. Montemor et al. [5] have observed that fly ash concrete offered good resistance to corrosion of reinforcement which is a very vital problem faced in reinforced concrete structures.

Al-Jabri et al. [6] investigated the effect of using copper slag as a substitute for fine aggregate in concrete. It has been observed by them that addition of copper slag improved the density of the concrete together with rapid increase in the workability. Also, they have noted that up to 40% replacement of fine aggregate by copper slag would yield High Performance Concrete with good strength and durability. Al-Jabri et al. [7] also have studied the effect of superplasticizer in concrete containing copper slag as substitute for fine aggregate. It was observed by them that while the compressive strength of concrete increased with the increase in the copper slag content, with the absence of superplasticizer the strength and durability of concrete decreased drastically. It was concluded that superplasticizer is very important in concrete containing copper slag as a replacement material for fine aggregate. Al-Jabri et al. [8] have also examined the effect on the compressive strength of mortar made with fine aggregate replaced with copper slag and found the compressive strength of the mortar to be comparable or higher than that of the control mix.

Song et al. [9] have examined the permeability characteristics of concrete containing silica fume. They have found that the permeability of concrete decreases rapidly when the silica fume content exceeds 8% of cement content. Dotto et al. [10] investigated the effect of addition of silica fume on physical properties of concrete and corrosion of reinforcement. They showed that the compressive strength is improved and addition of 6% of silica fume increased the resistance to reinforcement corrosion by 2.5 times. Mazloom et al. [11] have studied the short-term and long-term mechanical properties of high-strength concrete containing different levels of silica fume. They have observed that increasing the quantity of silica fume reduced workability, decreased expansion and basic creep, and increased 28-day compressive strength and secant modulus. While the addition of silica fume does not significantly affect total shrinkage, autogenous shrinkage increased as the amount of silica fume increased.

Ding and Kusterle [12] have observed an interesting observation on the behavior of concrete containing steel fibres. They have found that the presence of steel fibres increases the compressive strength significantly at the earlier age even though no significant improvement is found in the strength.

While studies reported are large in number with respect to using these wastes as materials to replace cement and aggregates in concrete making, it is noted from the studies made so far that these studies have been carried out either with cement being replaced by fly ash/silica fume or fine/coarse aggregates being replaced by fly ash/copper slag. The studies are very scarce which deal with replacing both cement and aggregates with suitable substitute. In the present study, the combination of fly ash, silica fume, and copper slag as partial replacement is used for both cement and fine aggregate. Since superplasticizer is a must to be used if copper slag is used as substitute for fine aggregate [7], it is used in concrete making. Creep is one of the major problems faced in large concrete girder. Since silica fume has the property to reduce basic creep [11] of concrete, fixed percentage of silica fume has been added to the concrete containing fly ash and copper slag. Steel and polypropylene fibres have been added to the mix to study its effect also. Though addition of fibres would not produce any significant effect on the compressive strength, it will manifest itself in improving the early age strength [12], tensile strength, and therefore flexural strength.

## 2. Scope of the Study

The scope of the present study is to determine

- the effect of addition of fly ash, copper slag, steel fibres, and polypropylene fibres on compressive strength of concrete
- the hierarchical order of influence of variables or “objects” affecting the compressive strength of concrete using cluster analysis in MATLAB.

The target compressive strength for the control specimen is chosen as 58 MPa. While fly ash and copper slag are used for partial replacement of cement and fine aggregate, respectively, defined quantity of silica fume, steel fibres, and polypropylene fibres is added to the mix proportions considered for the study.

## 3. Experiments

### 3.1. Materials Used and Testing Details

Ordinary Portland cement, locally available River sand, and crushed granites (of size 20 mm) have been used for preparing concrete. Concrete cubes of size 150 × 150 mm are cast in a controlled environment in the moulds. The moulds are dismantled after 24 hours and the specimens are subjected to immerse curing for 28 days. After the curing period, the cubes are tested for their compressive strength. Mix proportions considered for preparing the concrete are given in Table 1.

It can be seen from Table 1 that the amount of cement replaced by fly ash is varied from 40% to 60% (by weight). For each percentage of replacement of cement, the amount of fine aggregate replaced by copper slag is varied from 30% to 50% (by weight). Amount of silica fume and water-binder ratio are kept constant as 6% of cement and 0.35, respectively, for all the specimens. Amount of superplasticizer varied from 2.0 to 2.2% by weight of the binder content. Amounts of steel fibres and polypropylene fibres added are based on the percentage of volume of binder content. The percentage is 0.25% when both fibres are present and 0.5% when any one type of fibre is present.

### 3.2. Analysis of Results

A total of 37 mixes (including control mix) were considered for the experimental study. Three cubes were cast for a given mix. The minimum, maximum, and the average of the three compressive strengths determined for a given mix are reported in the Table 2 and the results are presented graphically in Figures 1, 2, 3, 4, 5, 6, 7, 8, 9, 10, and 11.

Table 3 gives the relation between FA-C and CS-S and their corresponding percentages of replacement of cement and fine aggregate, respectively.

### 3.3. Inferences Made without Considering the Presence of Steel and Polypropylene Fibres

**Figure 1:**
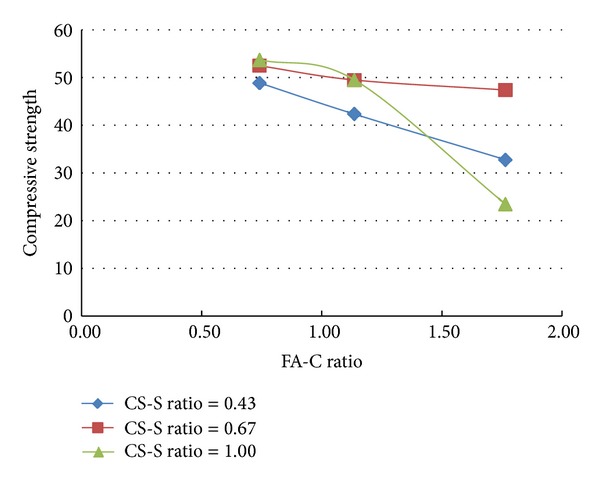
Compressive strength versus FA-C ratio (specimens S1–S9: without fibres).

**Figure 2:**
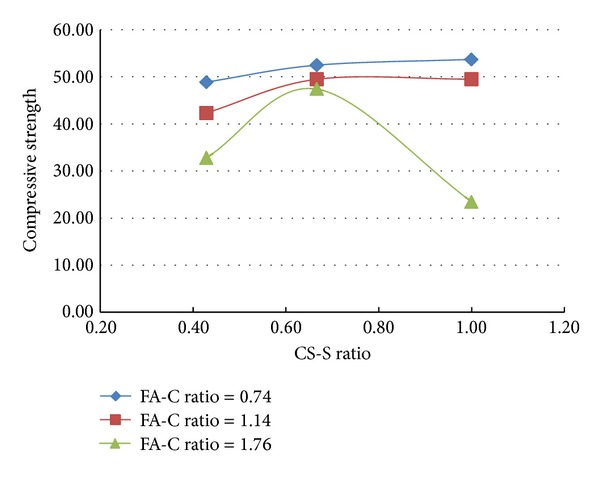
Compressive strength versus CS-S ratio (specimens S1–S9: without fibres).

It is inferred from Figures 1 and 2 that, in general,

- for a given copper slag-fine aggregate ratio, increase in fly ash-cement ratio decreased the strength of the concrete and the rate of decrease in the strength decreased with increase in copper slag-sand ratio,
- for a given fly ash-cement ratio, increase in copper slag-fine aggregate ratio increased the strength of the concrete,
- for a given copper slag-fine aggregate ratio, the strength of concrete seems to vary linearly with variation in fly ash-cement ratio and for a given fly ash-cement ratio, the strength of concrete seems to vary nonlinearly with variation in copper slag-fine aggregate. It is also interesting to note that, as long as both ratios (namely, fly ash cement or copper slag sand) are not *≈*≥1.0, there seems to be no abrupt change in the trend of variation of strength.

### 3.4. Inferences Considering the Presence of Steel and Polypropylene Fibres

From the results of experiments presented in Table 2 and the Figures 3–11, the following inferences are made.

- Generally, for all the specimens considered for this study, it is noted that the addition of steel fibres increased the strength by about 3% on the average, but addition of polypropylene fibres decreased the strength by about 3% on the average.
- Additions of polypropylene fibres seem to affect the linear variation of strength with variation in fly ash-cement ratio. The nonlinearity in the relation between strength of concrete and variation of fly ash-cement ratio seems to be higher for higher copper slag-fine aggregate ratio.
- A much interesting observation is that, when the fly ash-cement ratio was 1.76 and copper slag-sand ratio was 1.0,
  - when compared to the mix where *no fibres* are present,
    - in the presence of *only steel fibres*, compressive strength increased by 12%,
    - in the presence of *only polypropylene fibres*, compressive strength increased by 21%,
    - in the presence of *both steel and polypropylene fibres*, compressive strength increased by 53%,
  - when compared to the control mix, it is found that the addition of 0.25% of both steel and polypropylene fibres has lowered the strength reduction from 56.9% to 33.9%.
- Irrespective of the presence of fibres, the variation in the strength (with respect to control mix) was found to be within ±7% when the fly ash used was 40% and copper slag used was 50%.

**Figure 12:**
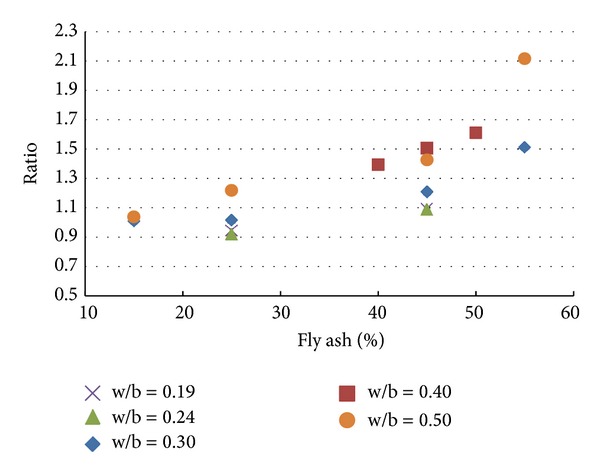
Plot of experimental results from [10, 16, 17].

To understand the effect of replacement of cement by fly ash without using any substitute for any other ingredients for making concrete, the results of the experiments conducted are compared with Poon 2009, [2, 13]. The results taken are tabulated in Table 4 and the same are plotted in a useful form in Figure 12.

**Figure 13:**
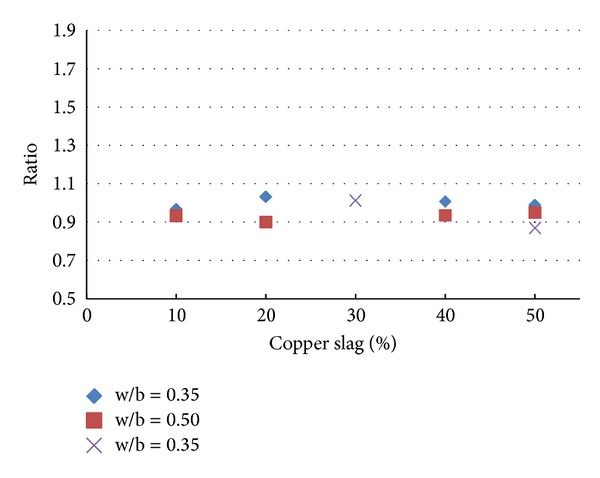
Results of experiment with no fibres.

Also, to understand the effect of replacement of fine aggregate by copper slag without using substitute for any other ingredients for making concrete, results of the experiments are taken from [6–8]. The results taken are tabulated in Table 5 and the same are plotted in a useful form in Figure 13.

It is inferred from Figures 12 and 13 that, for the range of fly ash and copper slag replacement levels considered for the present study, the strength behaviour of concrete containing copper slag as replacement of fine aggregate is relatively lesser sensitive to the water-binder ratio than that of concrete containing fly ash as substitute for cement. In the water-binder ratio range 0.35–0.50, the variation of strength is observed to be large in concrete containing fly ash as substitute for cement than in concrete containing copper slag as substitute for fine aggregate.  Table 6 gives the ratios (as determined in Tables 4 and 5) determined using the experimental study reported in this paper.

**Figure 14:**
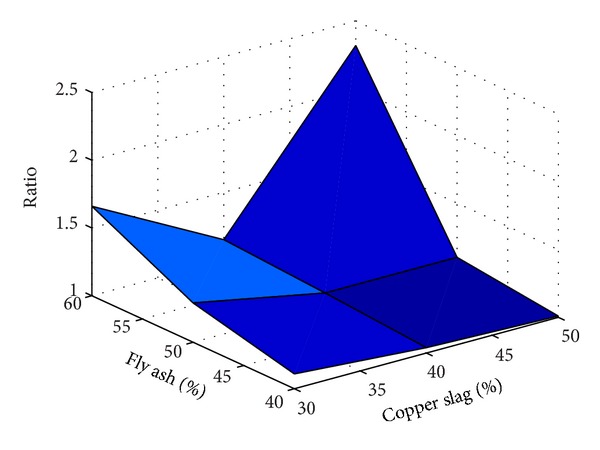
Results of experiment with steel fibres.

**Figure 15:**
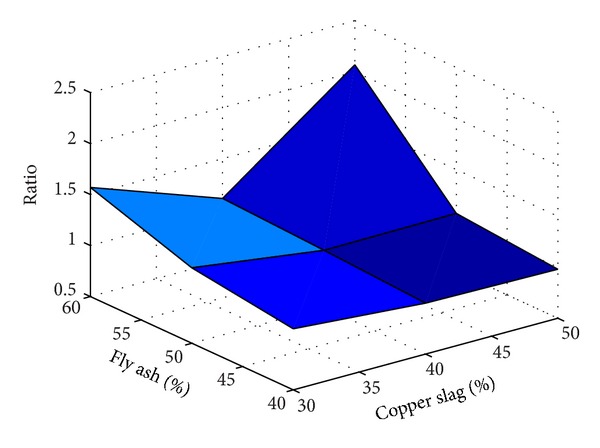
Results of experiment with polypropylene fibres.

**Figure 16:**
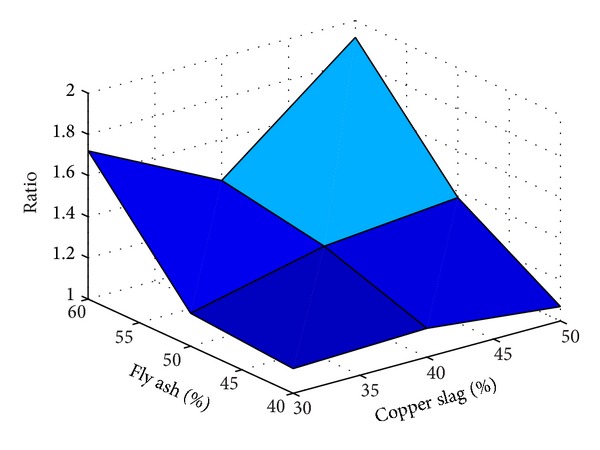
Results of experiment with steel and polypropylene fibres.

**Figure 17:**
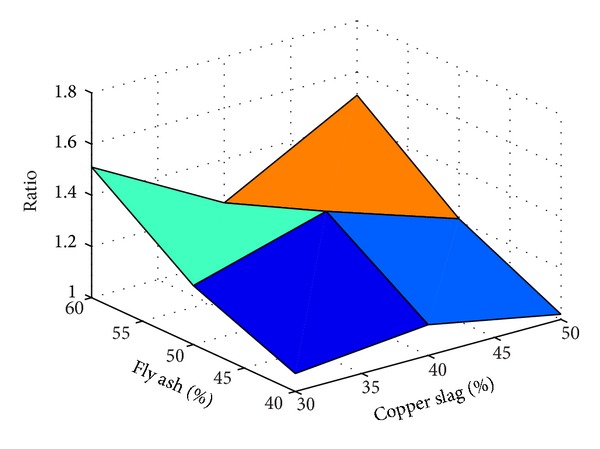
Typical dendrogram [18].

It is observed from Table 6 that, for 30% replacement of fine aggregate with copper slag, for all the percentages of fly ash considered, the ratios determined vary largely irrespective of the presence of fibres and type of fibres present. But the variation has reduced considerably when 40% of fine aggregate is replaced by copper slag. As noted earlier, this observation reinforces the statement that it seems to be *necessary* to use 40% copper slag to replace the fine aggregate in order to achieve comparable strength as that of control concrete. A similar observation is made for 50% use of copper slag but the observation is valid for only 40% and 50% usage of fly ash. The ratio is increased when the fly ash percentage was 60%. While the presence/absence of steel fibres does not produce significant effect in the strength, presence of polypropylene fibres reduced the strength. The results tabulated in Table 6 are plotted in Figures 14, 15, 16, and 17.

From Figures 14–17, it is seen that while the presence of steel fibres does not alter the behavior of concrete with respect to the strength, polypropylene does alter it. These figures would help to understand the variation of the strength of concrete observed in this study with different percentages of fly ash and copper slag as partial replacement for cement and fine aggregate, respectively.

## 4. Cluster Analysis

Analysing large quantity of data can be easily performed using cluster analysis. Cluster analysis deals with dividing the data into groups based on the information found on the data (http://www-users.cs.umn.edu). The two kinds of clustering that can be performed in MATLAB are

1. hierarchical clustering,
2. K-means clustering.

In the year 1967, Johnson [14] introduced the method of Hierarchical clustering which helps in identifying “groups” or “clusters” in large group of data. In this method, the clusters are formed using different “objects” based on the “similarities” found on the data. The “similarity” is determined by the “Euclidean distance” between the “objects.” The method of clustering first assumes that all the “objects” considered are individual clusters by themselves. Then by determining the “similarities” between the “objects,” numbers of clusters with different “objects” are formed. This process of clustering different “objects” continues until all the “objects” considered together form one “strong cluster.” By this method, one can get the real “feel” for what the clusters tell us, which is very vital.

In K-means clustering, invented by MacQueen in the year 1967 [15],  a *required* number of partitions or “clusters” are formed on the data. In this method, the centroid of the cluster is formed by choosing randomly. As far as possible the centroids should be apart from each other because different positions of the centroids yield different results. Once the centroids are chosen, the data points are “associated” with the centroids near to it and the process continued until all the data points are associated with any of the centroids defined. Then the centroids of the clusters thus have formed and are recalculated as barycentres of the data points associated with the clusters and the procedure was repeated until no changes in the positions of the centroids are observed.

**Figure 18:**
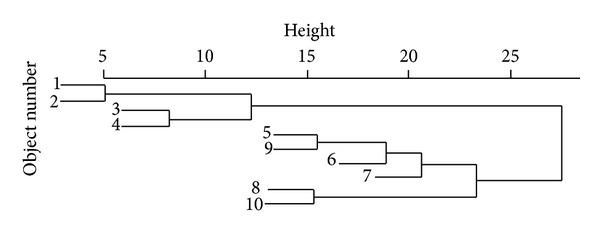
Dendrogram obtained.

Since our aim is to determine the “order of influence” of the mix variables which affect the sustainable concrete strength. the hierarchical cluster analysis is used to determine the hierarchical order of influence of variables or “objects” (S-C, CA-C, FA-C, CS-C, SF-C, S_fibres-C, P_fibres-C, w-b) on compressive strength (another “object”) of concrete containing fly ash, copper slag, silica fume and fibres. The results of the cluster analysis are shown by “dendrogram”, a cluster-tree.  Figure 18 shows a qualitative measure of the distance at which the “objects” are “linked” as in [16].

### 4.1. Inferences Made

**Figure 19:**
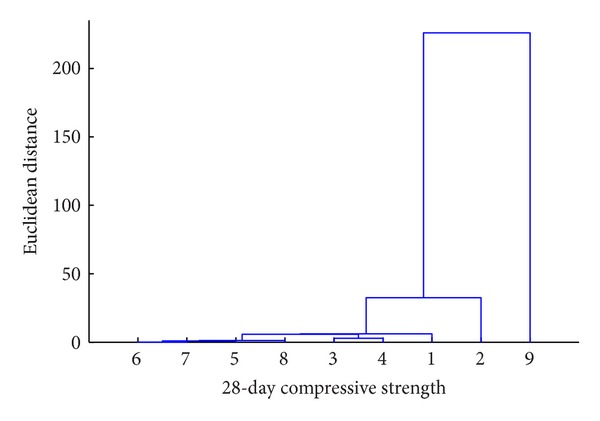
Dendrogram obtained (with portions enlarged).

**Figure 20:**
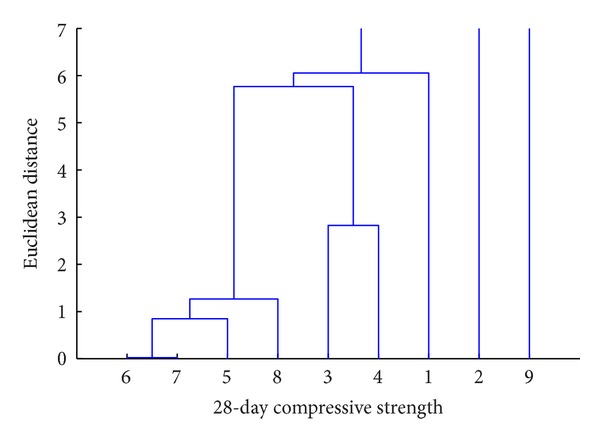
Plot of experimental results from [6, 8, 9].

It can be seen from Figure 18 that the “objects” 1 and 2 are linked to each other at a distance of 5 units to form cluster-1 and the “objects” 3 and 4 are linked at a distance of around 8 units to form cluster-2. These two clusters are linked together at a distance of around 12 units. It is inferred from the dendrogram that the “objects” 1 and 2 are “closely linked to each other” and similarly, 3 and 4 are. The clusters formed by “objects” 1 and 2 and 3 and 4 together form another cluster (say cluster-3) which is linked, at a higher distance, to another cluster formed in a similar way. It is this technique that is used in this study to determine the hierarchical influence of S/C, CA/C, FA/C, CS/C, SF/C, S_fibres/C, P_fibres/C, and w-b on strength.  Table 7 shows the “objects” considered and the corresponding object number. The results of the cluster analysis are presented in Figures 19 and 20.

From the dendrogram shown in Figure 19, it is observed that the “object-9” (strength) is first connected to the link from the “object-2” (CA-C). It means that CA-C ratio predominantly affects the strength than any other variable. From Figure 20, it is noted that next to CA-C is the “object-1” (S-C) that is linked to the “object-9.” Next to S-C, the “object-3 and object-4” (FA-C and CS-C) are found joined together to connect to the “object-9.” The “object-3 and object-4” are followed by the “object-8 and object-5” (w-b and SF-C). At last the “object-6 and object-7” (S_fibres/C and P_fibres/C) affect the strength.

The quantity of coarse aggregate, present in the concrete, that predominantly affects its strength may be attributed, due to this reason the coarse aggregate is only ingredient in the concrete which has strength even in its “raw material” state. The fine aggregate quantity which forms a major part together with the cement in holding the coarse aggregate forms the next “object” in affecting the strength. It is interesting to note that the presence of fly ash and copper slag, by linking to the “object-9” together, means that both these “objects” have equal influence in dictating the strength of the concrete, whatever may their percentage of replacing be. Even though fly ash and copper slag have equal influence in the strength, it is reiterated here that the replacement of fine aggregates at different percentages by copper slag produces lesser variation in the strength than the strength variation produced when the cement is replaced at different percentages by fly ash. Fly ash and copper slag quantities are followed by water-binder ratio which is then followed by silica fume content in affecting the concrete strength. It seems that since the quantity of silica fume has been kept constant for all the mix proportions, it has been shifted to the last in the hierarchical list obtained from the cluster analysis. To reinforce that the presence of the fibres affects the strength only marginally, it is inferred from the dendrograms that the presence of both fibres is linked at the last to the “object” strength.

## 5. Conclusion

The following conclusions are drawn from the analysis carried out.

1. If fly ash is used for partial replacing cement in concrete making, it seems to be necessary to replace fine aggregate *also* by copper slag, ensuring that not both ratios (namely, fly ash cement or copper slag sand) exceed 1.0.
2. Addition of both steel and polypropylene fibres to the mix seems to reduce the decrease in the strength when both ratios (namely, fly ash cement or copper slag sand) are *≈*≥1.0.
3. Replacing 40% of cement by fly ash and 50% of fine aggregate by copper slag (with or without fibres) seems to be the optimum replacement percentages to achieve comparable strength to that of the control mix.
4. Cluster analysis revealed that both fly ash and copper slag have equal influence in affecting the strength of the concrete whatever may their percentage of replacing be.

## Figures and Tables

**Figure 3:**
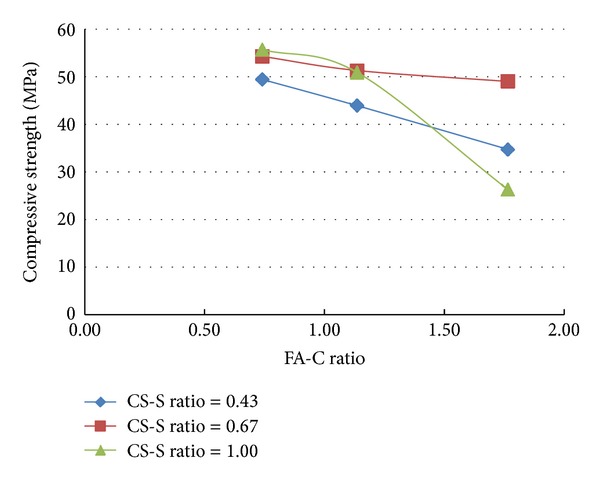
Compressive strength versus FA-C ratio (specimens S10–S18: with steel fibres).

**Figure 4:**
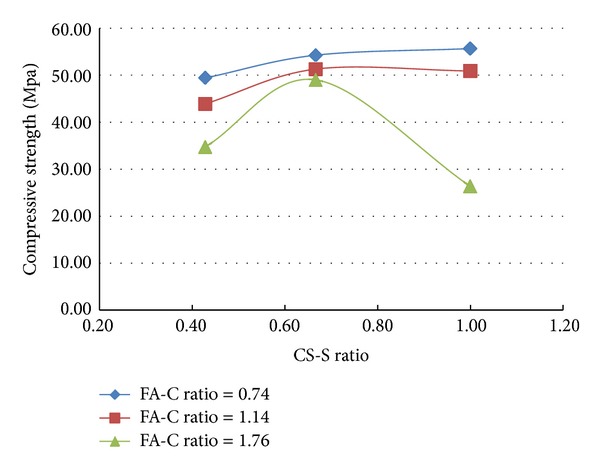
Compressive strength versus CS-S ratio (specimens S10–S18: with steel fibres).

**Figure 5:**
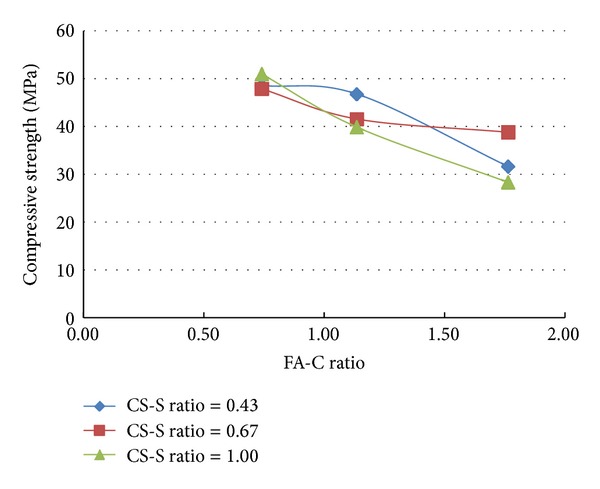
Compressive strength versus FA-C ratio (specimens S19–S27: with polypropylene fibres).

**Figure 6:**
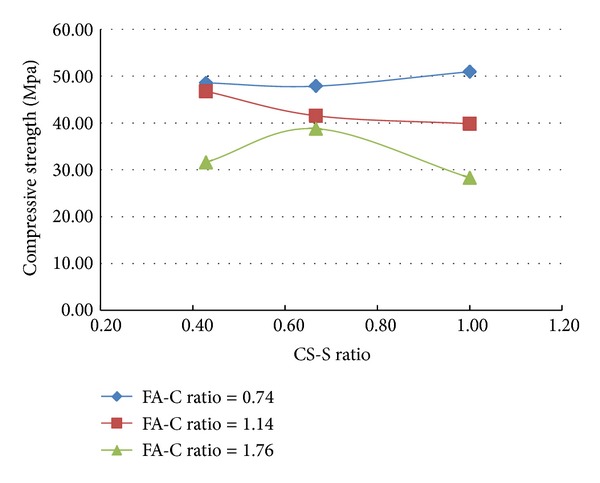
Compressive strength versus CS-S ratio (specimens S19–S27: with polypropylene fibres).

**Figure 7:**
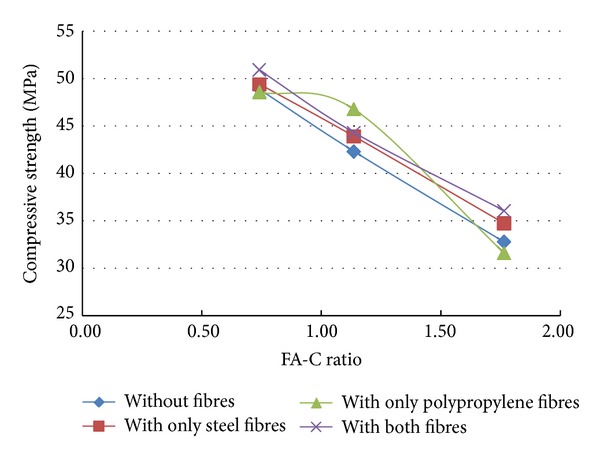
Effect of addition of fibres (CS-S ratio = 0.43).

**Figure 8:**
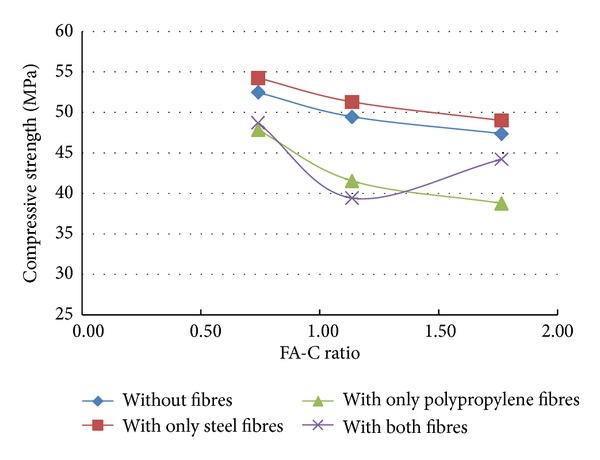
Effect of addition of fibres (CS-S ratio = 0.67).

**Figure 9:**
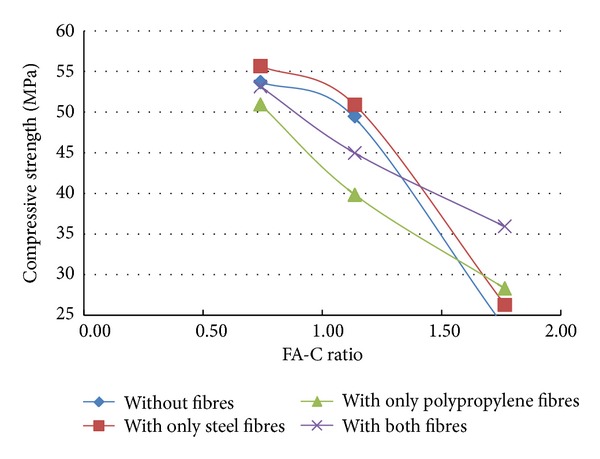
Effect of addition of fibres (CS-S ratio = 1.0).

**Figure 10:**
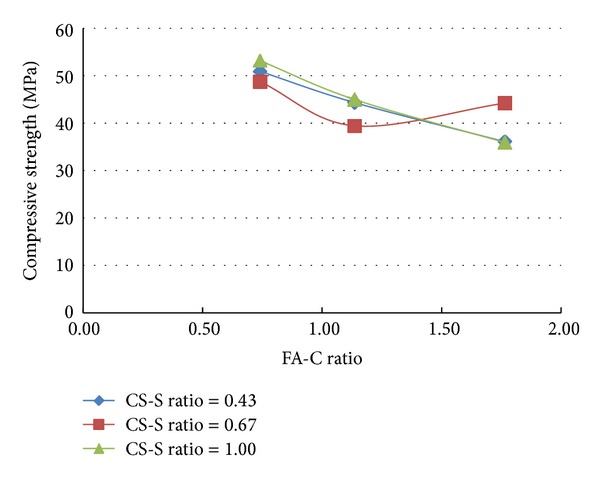
Compressive strength versus FA-C ratio (specimens S28–S36: with both fibres).

**Figure 11:**
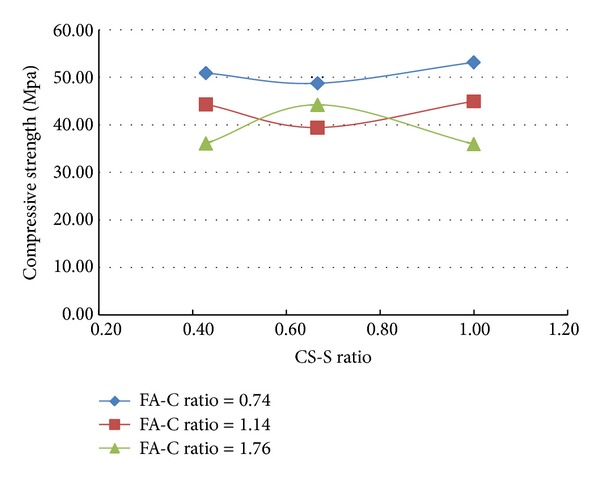
Compressive strength versus CS-S ratio (specimens S28–S36: with both fibres).

**Table 1:** Mix proportions (in kg/m^3^) considered for experimental study.

Mix ID	Cement	Fly ash	Silica fume	Fine aggregate	Copper Slag	Coarse aggregate	Steel fibre	Polypropylene Fibre
S0	400	—	—	652.0	—	1294	—	—
S1	216	160	24	456.4	195.6	1294	—	—
S2	176	200	24	456.4	195.6	1294	—	—
S3	136	240	24	456.4	195.6	1294	—	—
S4	216	160	24	391.2	260.8	1294	—	—
S5	176	200	24	391.2	260.8	1294	—	—
S6	136	240	24	391.2	260.8	1294	—	—
S7	216	160	24	326.0	326.0	1294	—	—
S8	176	200	24	326.0	326.0	1294	—	—
S9	136	240	24	326.0	326.0	1294	—	—
S10	216	160	24	456.4	195.6	1294	2	—
S11	176	200	24	456.4	195.6	1294	2	—
S12	136	240	24	456.4	195.6	1294	2	—
S13	216	160	24	391.2	260.8	1294	2	—
S14	176	200	24	391.2	260.8	1294	2	—
S15	136	240	24	391.2	260.8	1294	2	—
S16	216	160	24	326.0	326.0	1294	2	—
S17	176	200	24	326.0	326.0	1294	2	—
S18	136	240	24	326.0	326.0	1294	2	—
S19	216	160	24	456.4	195.6	1294	—	2
S20	176	200	24	456.4	195.6	1294	—	2
S21	136	240	24	456.4	195.6	1294	—	2
S22	216	160	24	391.2	260.8	1294	—	2
S23	176	200	24	391.2	260.8	1294	—	2
S24	136	240	24	391.2	260.8	1294	—	2
S25	216	160	24	326.0	326.0	1294	—	2
S26	176	200	24	326.0	326.0	1294	—	2
S27	136	240	24	326.0	326.0	1294	—	2
S28	216	160	24	456.4	195.6	1294	1	1
S29	176	200	24	456.4	195.6	1294	1	1
S30	136	240	24	456.4	195.6	1294	1	1
S31	216	160	24	391.2	260.8	1294	1	1
S32	176	200	24	391.2	260.8	1294	1	1
S33	136	240	24	391.2	260.8	1294	1	1
S34	216	160	24	326.0	326.0	1294	1	1
S35	176	200	24	326.0	326.0	1294	1	1
S36	136	240	24	326.0	326.0	1294	1	1

**Table 2:** Compressive strength (28-day) of specimens.

S. No.	Min	Max	Avg.
S0	52.9	56.8	54.4
S1	47.9	49.8	48.9
S2	40.9	43.8	42.3
S3	32.0	33.6	32.8
S4	42.8	57.8	52.5
S5	43.8	52.8	49.5
S6	46.6	48.8	47.4
S7	52.7	54.8	53.7
S8	47.2	51.0	49.5
S9	22.0	24.8	23.5
S10	46.7	51.8	49.4
S11	41.3	46.0	43.9
S12	34.2	35.8	34.7
S13	44.4	59.7	54.3
S14	45.8	54.3	51.3
S15	48.0	50.1	49.0
S16	54.7	56.8	55.7
S17	48.9	52.7	50.9
S18	24.4	27.8	26.3
S19	46.7	50.1	48.6
S20	45.8	48.3	46.7
S21	30.2	32.6	31.6
S22	43.6	51.1	47.9
S23	40.0	42.8	41.5
S24	37.8	40.3	38.8
S25	48.9	52.8	50.9
S26	31.1	45.3	39.8
S27	24.9	30.7	28.3
S28	49.1	53.3	50.9
S29	40.0	47.2	44.3
S30	33.8	38.0	36.1
S31	47.1	50.2	48.7
S32	35.6	42.7	39.4
S33	41.3	46.4	44.2
S34	51.7	54.4	53.1
S35	43.6	46.9	44.9
S36	33.3	38.0	35.9

**Table 3:** FA-C and CS-S ratios in terms of % of materials replaced.

FA-C ratio	FA in % of Cement	CS-S ratio	CS in % of Fine Aggregate
0.74	40	0.43	30
1.14	50	0.67	40
1.76	60	1.00	50

**Table 4:** Experimental results from the references (Poon 2009, [2, 13]).

Reference	% of FA	28-day compressive strength, N/mm^2^	Ratio	w/b
[8]	Control mix	37.2	—	0.41
	40	26.7	1.00	0.40
	45	24.7	1.08	0.41
	50	23.1	1.16	0.40

	Control mix	97.4	—	0.24
	25	105.9	0.92	0.24
[6]	45	89.4	1.09	0.24
	Control mix	96.8	—	0.19
	25	102.3	0.95	0.19
	45	88.5	1.09	0.19

	Control mix	86.8	—	0.30
	15	86.0	1.01	0.30
	25	85.4	1.02	0.30
	45	71.8	1.21	0.30
[9]	55	57.4	1.51	0.30
	Control concrete	50.8	—	0.50
	15	48.9	1.04	0.50
	25	41.7	1.22	0.50
	45	35.6	1.43	0.50
	55	24.0	2.12	0.50

Ratio = Compressive strength of control mix/compressive strength of fly ash concrete.

**Table 5:** Experimental results from the references ([6–8]).

Reference	% of CS	28-day compressive strength, N/mm^2^	Ratio	w-b
	Control mix	36.2	—	0.50
	10	38.8	1.00	0.50
	20	40.2	0.97	0.50
[17]	40	38.7	1.00	0.50
	50	38.1	1.02	0.50
	60	37.7	1.03	0.50
	80	27.8	1.40	0.50
	100	29.0	1.34	0.50

	Control mix	88.1	—	0.35
	30	87.1	1.00	0.32
[16]	50	101.3	0.86	0.31
	70	104.4	0.83	0.30
	80	101.6	0.86	0.29
	100	107.4	0.81	0.27

	Control mix	76.9	—	0.35
	10	79.6	1.00	0.35
	20	74.5	1.07	0.35
[10]	40	76.4	1.04	0.35
	50	77.8	1.02	0.35
	60	69.0	1.15	0.35
	80	63.8	1.25	0.35
	100	63.4	1.26	0.35

Ratio = Compressive strength of control mix/compressive strength of concrete containing copper slag.

**Table 6:** Experimental results from the present study.

	Mix ID	Strength	Ratio	Mix ID	Strength	Ratio	Mix ID	Strength	Ratio	Mix ID	Strength	Ratio	
30% of CS	S1	48.87	1.11	S10	49.42	1.10	S19	48.56	1.12	S28	50.89	1.07	40% of FA
	S2	42.32	1.29	S11	43.91	1.24	S20	46.76	1.16	S29	44.31	1.23	50% of FA
	S3	32.77	1.66	S12	34.74	1.57	S21	31.60	1.72	S30	36.07	1.51	60% of FA

40% of CS	S4	52.46	1.04	S13	54.26	1.00	S22	47.85	1.14	S31	48.72	1.12	40% of FA
	S5	49.47	1.10	S14	51.30	1.06	S23	41.54	1.31	S32	39.42	1.38	50% of FA
	S6	47.38	1.15	S15	49.00	1.11	S24	38.78	1.40	S33	44.22	1.23	60% of FA

50% of CS	S7	53.69	1.01	S16	55.66	0.98	S25	50.94	1.07	S34	53.14	1.02	40% of FA
	S8	49.48	1.10	S17	50.89	1.07	S26	39.83	1.37	S35	44.96	1.21	50% of FA
	S9	23.45	2.32	S18	26.30	2.07	S27	28.30	1.92	S36	35.92	1.51	60% of FA

	No fibres			0.5% of steel fibres			0.5% of polypropylene fibres			0.25% of steel and polypropylene fibres			

Ratio = Compressive strength of control concrete (54.40)/compressive strength of concrete containing fly ash and copper slag.

**Table 7:** Objects and Object number.

Object No.	1	2	3	4	5	6	7	8	9

Variable	S-C	CA-C	FA-C	CS-C	SF-C	S_fibre-C	P_fibre-C	w-b	28-compressive strength

## Notations

S-C: Sand-cement ratio
CA-C: Coarse aggregate-cement ratio
FA-C: Fly ash-cement ratio
CS-C: Copper slag-cement ratio
CS-S: Copper slag-fine aggregate ratio
SF-C: Silica fume-cement ratio
S_fibres-C: Steel fibres-cement ratio
P_fibres-C: Polypropylene fibres-cement ratio
w-b: Water-binder ratio.
